# Expression of interferon-regulated genes in juvenile dermatomyositis versus Mendelian autoinflammatory interferonopathies

**DOI:** 10.1186/s13075-020-02160-9

**Published:** 2020-04-06

**Authors:** Hanna Kim, Fatima Gunter-Rahman, John A. McGrath, Esther Lee, Adriana A. de Jesus, Ira N. Targoff, Yan Huang, Terrance P. O’Hanlon, Wanxia L. Tsai, Massimo Gadina, Frederick W. Miller, Raphaela Goldbach-Mansky, Lisa G. Rider

**Affiliations:** 1 Pediatric Translational Research Branch, National Institute of Arthritis and Musculoskeletal and Skin Diseases, NIH, Bethesda, MD USA; 2 Environmental Autoimmunity Group, Clinical Research Branch, National Institute of Environmental Health Sciences, NIH, Bethesda, MD USA; 3 Social & Scientific Systems, Inc., Durham, NC USA; 4 Translational Autoinflammatory Diseases Section, National Institute of Allergy and Infectious Diseases, NIH, Bethesda, MD USA; 5 VA Medical Center, University of Oklahoma Health Sciences Center, and Oklahoma Medical Research Foundation, Oklahoma City, OK USA; 6 Translational Immunology Section, National Institute of Arthritis and Musculoskeletal and Skin Diseases, NIH, Bethesda, MD USA

**Keywords:** (3–10): juvenile dermatomyositis, Myositis, Pediatric rheumatology, Interferon, Biomarkers, Interferonopathy, Myositis-specific autoantibodies

## Abstract

**Background:**

Juvenile dermatomyositis (JDM) is a systemic autoimmune disease with a prominent interferon (IFN) signature, but the pathogenesis of JDM and the etiology of its IFN signature remain unknown. The Mendelian autoinflammatory interferonopathies, Chronic Atypical Neutrophilic Dermatosis with Lipodystrophy and Elevated temperature (CANDLE) and STING-Associated Vasculopathy with onset in Infancy (SAVI), are caused by genetic mutations and have extremely elevated IFN signatures thought to drive pathology. The phenotypic overlap of some clinical features of CANDLE and SAVI with JDM led to the comparison of a standardized interferon-regulated gene score (IRG-S) in JDM and myositis-specific autoantibody (MSA) JDM subgroups, with CANDLE and SAVI.

**Methods:**

A peripheral 28-component IRG-S assessed by NanoString™ in 57 JDM patients subtyped by MSA was compared with IRG-S in healthy controls (HC) and CANDLE/SAVI patients. Principal component analysis (PCA) was performed, and individual genes were evaluated for their contribution to the score. IRG-S were correlated with disease assessments and patient characteristics.

**Results:**

IRG-S in JDM patients were significantly higher than in HC but lower than in CANDLE or SAVI. JDM IRG-S overlapped more with SAVI than CANDLE by PCA. Among MSA groups, anti-MDA5 autoantibody-positive patients’ IRG-S overlapped most with SAVI. The *IFI27* proportion was significantly higher in SAVI and CANDLE than JDM, but *IFIT1* contributed more to IRG-S in JDM. Overall, the contribution of individual interferon-regulated genes (IRG) in JDM was more similar to SAVI. IRG-S correlated moderately with JDM disease activity measures (*r*_*s*_ = 0.33–0.47) and more strongly with skin activity (*r*_*s*_ = 0.58–0.79) in anti-TIF1 autoantibody-positive patients. Weakness and joint disease activity (multinomial OR 0.91 and 3.3) were the best predictors of high IRG-S.

**Conclusions:**

Our findings demonstrate peripheral IRG expression in JDM overlaps with monogenic interferonopathies, particularly SAVI, and correlates with disease activity. Anti-MDA5 autoantibody-positive JDM IRG-S were notably more similar to SAVI. This may reflect both a shared IFN signature, which is driven by IFN-β and STING pathways in SAVI, as well as the shared phenotype of vasculopathy in SAVI and JDM, particularly in anti-MDA5 autoantibody-positive JDM, and indicate potential therapeutic targets for JDM.

## Background

Juvenile dermatomyositis (JDM) is a complex autoimmune disease characterized by weakness and rashes [1]. Myositis-specific autoantibodies (MSA) define phenotypic features and prognosis within JDM, with anti-TIF1, anti-NXP2, and anti-MDA5 autoantibodies being the most common MSA groups in JDM [1–4]. Although the etiology of JDM is presently unknown, multiple genetic and environmental factors contribute [1, 5]. Interferon (IFN)-regulated genes (IRG) are upregulated in the blood, muscle, and skin of patients with JDM and adult dermatomyositis (DM) and correlate with disease activity [6–13], although the exact source, mechanism, and role of IFN remain unclear and detailed assessments by MSA group are lacking.

Mendelian autoinflammatory interferonopathies, which are associated with a strong IRG signature, include Chronic Atypical Neutrophilic Dermatosis with Lipodystrophy and Elevated temperature (CANDLE) caused by additive loss-of-function mutations in proteasome components [14–16] and STING-Associated Vasculopathy with onset during Infancy (SAVI), resulting from gain-of-function mutations in the Stimulator of IFN genes (STING) protein [16, 17]. These conditions have a very strong IRG signature and blocking IFN signaling with a Janus kinase (JAK) inhibitor correlates with clinical improvement and IRG-S decrease in the majority of 18 CANDLE and SAVI patients, with 50% of CANDLE patients achieving persistent clinical remission [16, 18–20]. A direct comparison of JDM to the monogenic interferonopathies may provide insight into the role of IFN in JDM, particularly as CANDLE and SAVI share some clinical features with JDM. For example, CANDLE is also associated with lipodystrophy, joint contractures, and myositis, while SAVI has frequent vasculopathy including distal ulcerations and interstitial lung disease [1, 16, 21].

Utilizing a 28 IRG score (IRG-S), which was developed and validated as a biomarker in CANDLE and SAVI [22], we evaluated peripheral blood IRG expression in JDM. Given prominent IRG signatures and overlapping clinical features of JDM with conditions with IFN-driven pathogenesis based on genetic mutations (CANDLE and SAVI), we aimed to better characterize and understand the role of IFN in JDM and its MSA subgroups through direct comparison to patients with CANDLE and SAVI.

## Methods

### Patient selection

Subjects were enrolled in National Institutes of Health institutional review board-approved natural history studies (Table 1). Active JDM patients (*n* = 57) met probable or definite Bohan and Peter criteria [23]. CANDLE (*n* = 11) and SAVI (*n* = 7) patients were genetically defined and used as positive interferonopathy controls [18]. Neonatal-onset multisystem inflammatory disease (NOMID) patients (*n* = 18) served as autoinflammatory controls (IL-1 mediated), in addition to 26 healthy controls (HC), both of which were IFN-negative [22]. HC were not age- or gender-matched to JDM patients, as we previously did not find significant differences in IRG-S based on these variables [22]. All JDM patients consented to a NIH/NIEHS IRB-approved protocol. All CANDLE, SAVI, NOMID, and HC patients consented to a NIH/NIAID IRB-approved protocol (NCT02974595). MSAs were identified by validated immunoprecipitation and immunoblotting methods [24]. MSAs with adequate numbers of JDM patients for analysis included anti-p155/140 (TIF1) (*n* = 21), anti-MJ (NXP2) (*n* = 11), anti-MDA5 (*n* = 11) autoantibodies, and MSA-negative (*n* = 9). Clinical data are available to characterize features for 56 JDM patients.

**Table 1:** Demographics of each condition

Characteristics		Median [IQR] or *N* (%)				
		JDM (*n* = 57)	CANDLE (*n* = 11)	SAVI (*n* = 7)	NOMID (*n* = 18)	HC (*n* = 26)
Age at evaluation (year)		9.5 [5.9–13.2]	6.1 [5.2–15.7]	16.9 [6.1–18.1]	8.5 [5.3–17.1]	25.9 [9.7–39.4]
Gender	Female	34 (60)	5 (45)	3 (43)	7 (39)	17 (65)
Race	White	36 (63)	4 (36)	7 (100)	12 (67)	15 (58)
	Hispanic	4 (7)	4 (36)	0	3 (17)	8 (31)
	Black	3 (5)	2 (18)	0	1 (6)	1 (4)
	Multiple race	10 (18)	0	0	1 (6)	1 (4)
	Other	4 (7)	1 (9)	0	1 (6)	1 (4)
Disease duration (months)		10.7 [4.9–36.7]	NA	NA	NA	NA

Abbreviations: *IQR* interquartile range, *NA* not applicable, *JDM* juvenile dermatomyositis, *CANDLE* Chronic Atypical Neutrophilic Dermatosis with Elevated temperature, *SAVI* STING-Associated Vasculopathy with onset during Infancy, *NOMID* Neonatal-onset multisystem inflammatory disease, *HC* healthy controls

### Materials

From a single sample per patient of whole blood collected in PAXgene tubes (Qiagen, Germantown, MD), total RNA was extracted. NanoString Technologies™ (Seattle, WA) was used for gene expression analysis, with scores calculated as a sum of *Z*-scores for each of 28 IRGs [22]. Exploratory analysis (data not shown) of complete blood count parameters (white blood cell count as well as absolute neutrophil, lymphocyte, monocyte, eosinophil, and basophil counts) between JDM (*n* = 55) versus CANDLE (*n* = 10) and SAVI (*n* = 5) FDR corrected for multiple comparisons did not detect any significant differences, so no further normalization based on these counts was performed.

### Analysis methods

Analysis was performed using R version 3.5.0 (The R Foundation for Statistical Computing, Vienna, Austria, ISBN 3-9000-51-07-0, http://www.r-project.org), GraphPad Prism7 (GraphPad Software, San Diego, CA), JMP13 (SAS Institute, Cary, NC), or SYSTAT13 (Systat Software, San Jose, CA).

### Whole blood score comparisons

With JDM overall, IRG-S were compared to CANDLE, SAVI, NOMID, and HC, and FDR corrected for multiple comparisons using the Benjamini, Krieger, and Yekutieli method [25], with significant *q* values < 0.05. Exploratory analysis compared subgroups of JDM (highest quartile of JDM IRG-S (JDM-HQ), JDM IRG-S above HC, or a specific MSA group) to each other, autoinflammatory conditions and/or HC by Kruskal-Wallis tests, followed by Dunn’s multiple comparisons tests (uncorrected), with *p* < 0.05 considered significant.

### Principal component analysis

For principal component analysis (PCA) of JDM, JDM-HQ, and MSA subgroups with the autoinflammatory diseases and controls, all PCAs had adequate samples, as indicated by Kaiser-Meyer Olkin test (values ≥ 0.86) and Bartlett’s sphericity test (*p* values < 0.0001). Five unrotated PCAs were performed using normalized gene counts, each with autoinflammatory diseases and HC data, differentiated by whether they included all JDM patients or a MSA subgroup as follows: PCA-A (all JDM, CANDLE, SAVI, NOMID, HC), PCA-B (anti-MDA5 autoantibody-positive subgroup of JDM, CANDLE, SAVI, NOMID, HC), PCA-C (anti-NXP2 autoantibody-positive subgroup of JDM, CANDLE, SAVI, NOMID, HC), PCA-D (anti-TIF1 autoantibody-positive subgroup of JDM, CANDLE, SAVI, NOMID, HC), and PCA-E (MSA-negative subgroup of JDM, CANDLE, SAVI, NOMID, HC). One anti-TIF1 JDM patient with suspected viral bronchitis had outlying IRG expression (> 99th percentile) and principal component (PC) scores, and was removed from the analysis. The component loadings are the correlations of the individual gene normalized *Z*-scores with the given principal component. A stronger loading (e.g., greater than 0.40 or less than − 0.40) indicates a stronger relationship or contribution of that gene with that principal component. As with correlations, these can be negative or positive.

### Gene proportion analysis

The contribution of each of the 28 individual IRGs was compared to the total IRG *Z*-score, which was normalized so that no gene component was negative. Each gene’s proportion of the total normalized IRG-S in JDM-HQ was compared to that in CANDLE and SAVI. The analysis was limited to JDM-HQ (*n* = 14); this subgroup of JDM has an IRG-S in the same range as CANDLE and SAVI for a more equitable comparison of individual gene proportions. As our goal was to assess whether the pattern of dysregulation in JDM IRG-S was the same as in CANDLE and SAVI, we assessed gene proportions from scores in the same IRG-S range in order to limit differences due simply to different levels of overall dysregulation.

### Ratios related to NF-κB and IFN-γ

The expression level of the 3 IFN response genes (*CXCL10*, *GPB1*, *SOCS1*) that are regulated by STAT1 and have nuclear factor kappa B (NF-κB) binding sites are differentially regulated in some conditions with elevated IRG-S, distinguishing a subset of diseases from CANDLE and SAVI [26]. Also, as the contribution of different interferons can be variable, the expression level of 9 of the IFN response genes (*CXCL10*, *EPSTI1*, *IFIT1*, *IFIT2*, *IFIT3*, *ISG15*, *LY6E*, *SOCS1*, *USP18*) with a greater increase in expression when induced by IFN-γ than IFN-α, over all 28 genes, was assessed as this has also been described to be variable in other interferonopathies versus CANDLE and SAVI [26]. Both the ratio of 3 NF-κB-related genes compared to the other 25 IRGs (“NF-κB ratio”) and the latter ratio of 9 genes over 28 (“IFN-γ ratio”) [26] were calculated based on normalized counts with analysis as above in JDM patients with elevated scores or IRG-S above healthy control range [22] and described by MSA group versus CANDLE and SAVI.

### IFN score correlations in JDM

IRG-S were correlated by Spearman’s rank (*r*_*s*_) with core set disease activity and damage assessments in JDM patients overall and in patients with anti-TIF1 autoantibodies [27] (Table 2). IRG-S were also correlated with corticosteroid doses. IRG-S was compared between JDM patients receiving specific medications or not by the Mann-Whitney test and analyzed by the number of steroid-sparing medications received via Kruskal-Wallis and Dunn’s (uncorrected) tests.

**Table 2:** Spearman’s rank correlations between selected myositis disease measures and interferon-regulated gene (IRG) scores

		28 gene IRG score		Other IRG scores in JDM	
Assessment (*n* = 57) (range or upper limit of normal (ULN))	JDM overall median, IQR	JDM overall (*n* = 57)	Anti-TIF1 Ab (*n* = 20)*	Bilgic et al. score^‡^ [6]	Greenberg et al. score^§^ [7]
28 IRG score (*n* = 57)	40.0 [1.8–236.0]	40.0 [1.8–236.0]	41.7 [−4.4–174.2]	0.96**	0.99**
Physician global activity (0–10 VAS, *n* = 56)	2.2 [1.5–3.7]	0.39^||^	0.31	0.42^||^	0.39^||^
Total MMT (0–260, *n* = 36)	242 [228–252]	− 0.36^§^	− 0.13	− 0.38^§^	− 0.33
CMAS (0–52, *n* = 38)	43 [37–48]	− 0.26	− 0.22	− 0.21	− 0.18
CHAQ/HAQ (0–3.0, *n* = 42)	0.75 [0.25–1.375]	0.28	0.23	0.24	0.30
MDAAT (0–10 VAS, *n* = 39)					
Muscle	2.0 [1.0–2.9]	0.31	0.29	0.40^§^	0.30
Constitutional	1.3 [0.3–3.3]	0.09	0.21	0.09	0.11
Cutaneous	2.4 [1.1–3.8]	0.29	0.62^§^	0.33^§^	0.30
Pulmonary	0.6 [0.0–1.4]	0.23	0.44	0.29	0.21
Skeletal (joint)	0.9 [0.0–2.1]	0.35^§^	− 0.12	0.42^||^	0.36^§^
Extra-muscular activity	1.9 [1.2–3.2]	0.47^||^	0.76^||^	0.53^¶^	0.48^||^
(0–10 VAS, *n* = 40)					
Disease Activity Score (0–20, *n* = 39)	11 [9–13]	0.33^§^	0.58^§^	0.35§	0.33^§^
DAS skin (0–9, *n* = 40)	6 [5–7]	0.16	0.73^||^	0.18	0.19
DAS muscle (0–11, *n* = 39)	5 [3.75–7]	0.25	0.24	0.26	0.23
Muscle enzymes (U/L)					
CK (ULN 252 U/L, *n* = 56)	90 [52–170]	−0.09	− 0.18	− 0.11	− 0.07
Aldolase (ULN 7 U/L, *n* = 54)	7.5 [5.8–10.1]	0.24	0.18	0.25	0.24
AST (ULN 34 U/L, *n* = 56)	24 [18–33]	0.42^||^	0.24	0.38^||^	0.43^¶^
LDH (ULN 226 U/L, *n* = 56)	186 [161–214]	0.41^||^	0.27	0.39^||^	0.39^||^
Physician global damage (0–10, *n* = 46)	1.0 [0.4–2.0]	−0.05	0.04	−0.09	− 0.05
MDI total damage (0–110, *n* = 34)	4.0 [1.8–8.0]	0.16	0.37	0.15	0.17

Abbreviations: *IQR* interquartile range, *JDM* juvenile dermatomyositis, *anti-TIF1* subgroup of JDM positive for anti-TIF1 autoantibodies, *Ab* autoantibody, *VAS* visual analog scale, *MMT* manual muscle testing, *CMAS* Childhood Myositis Assessment Scale, *CHAQ*: Childhood Health Assessment Questionnaire, *HAQ* Health Assessment Questionnaire, *MDAAT* Myositis Disease Activity Assessment Tool, *DAS* Disease Activity Score, *CK* creatine kinase, *AST* aspartate aminotransferase, *LDH* lactate dehydrogenase, *PGD* Physician global damage, *MDI* Myositis Damage Index

^§^*p* ≤ 0.05, ^||^*p* ≤ 0.01, ^¶^*p* ≤ 0.001, **p ≤ 0.0001

*Proportionate numbers had assessments as JDM overall

^†^Bilgic et al. score: *IFIT1*, *IRF7*, and *ISG15* as in [6]

^‡^Greenberg et al. score: *EPSTI1*, *HERC5*, *IFI27*, *IFI44*, *IFI44L*, *IFI6*, *IFIT1*, *IFIT3*, *ISG15*, *MX1*, *OAS1*, *OAS3*, and *RSAD2* as in [7]

Two published IRG-S from DM patients, *IFIT1*, *IRF7*, and *ISG15* [6] and *EPSTI1*, *HERC5*, *IFI27*, *IFI44*, *IFI44L*, *IFI6*, *IFIT1*, *IFIT3*, *ISG15*, *MX1*, *OAS1*, *OAS3*, and *RSAD2* [7], were calculated by summing the *Z*-score of relevant genes for correlation with JDM disease assessments.

JDM disease activity and damage core measures [27] (Additional file 1: Table S1) were compared among IRG-S above and below 48.9, the 95th percentile of HC [22], using Mann-Whitney or Fisher’s exact tests. Myositis Disease Activity Assessment Tool (MDAAT) cardiovascular and gastrointestinal visual analog scales (VAS) were excluded, as few patients had activity in these systems. Non-redundant measures (*r*_*s*_ < 0.7) that differentiated high and low IRG-S were included in backward stepwise logistic regression to examine those associated (alpha < 0.15) with high IRG-S (> 48.9).

### Interferon-related protein correlation

IP-10 (CXCL10) as part of a Luminex assay (Bio-Rad, Hercules, CA) was assessed in serum or plasma of JDM patients available, to compare the IRG-S with IFN-signaling at the protein level by Spearman’s correlation.

## Results

### Description of clinical features

Clinical characteristics were also descriptively compared between disease groups based on medical record review. Our cohort of JDM patients demonstrates some clinical overlap with CANDLE and SAVI including the presence of myositis, certain cutaneous features, musculoskeletal and systemic features, and damage findings albeit with different frequencies (Additional file 1: Table S2). Cutaneous features have distinctive characteristics in each condition, specifically heliotrope, Gottron’s papules, photosensitivity, V-sign, and shawl sign rashes present in JDM, whereas panniculitis, nodular violaceous erythema, and annular plaques are present prominently in CANDLE, and cutaneous ulceration in SAVI. All of the JDM patients had proximal weakness while 8/10 (80%) of CANDLE and 1/4 (25%) of SAVI had myositis. All of the SAVI patients with available data (4/4, 100%) had interstitial lung disease versus 8/56 (14.3%) of JDM and none of the CANDLE patients. Of note, there is some variation within JDM by the MSA group as previously described [1–4]. The anti-TIF1 autoantibody JDM subgroup has more photosensitivity (18/20, 90.0%) and shawl sign rash (8/20, 55.0%) versus other groups in JDM. The anti-NXP2 autoantibody JDM subgroup has more notable myositis with MMT < 225/260 (2/5, 40%) and less photosensitivity (5/11, 45.5%), V-sign (1/11, 9.1%), and shawl sign (0/11, 0.0%). Both have less interstitial disease (0/20 and 0/11, 0.0%). The anti-MDA5 autoantibody JDM subgroup has less notable myositis with MMT <225/260 (0/8, 0.0%) and more cutaneous ulceration and interstitial lung disease (both 7/10, 70.0%) (Additional file 1: Table S2).

### Whole blood IRG score analysis

**Fig. 1:**
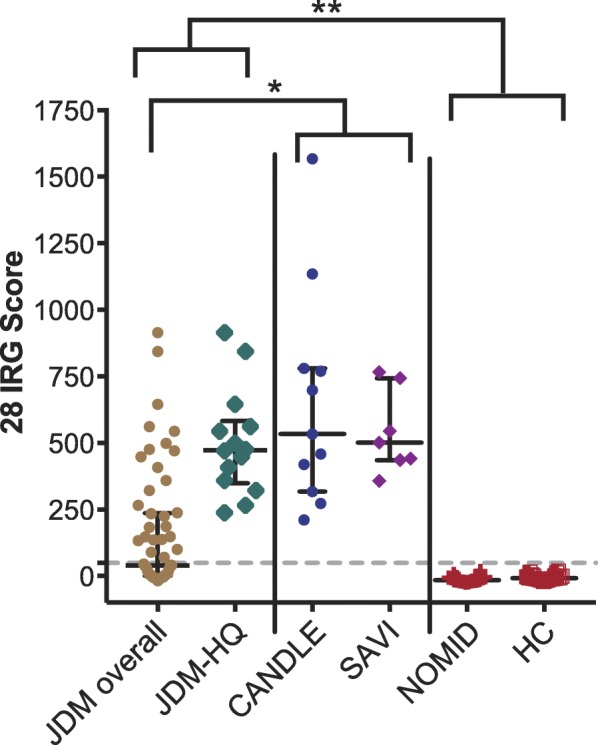
Peripheral blood interferon-regulated gene (IRG) scores in juvenile dermatomyositis (JDM) compared to monogenic autoinflammatory disease patients and healthy controls. Median and interquartile ranges are shown. Dotted horizontal line represents 95th percentile of healthy controls [22]. CANDLE (*n* = 11), SAVI (*n* = 7), NOMID (*n* = 18), HC (*n* = 26). JDM overall (*n* = 57):*q < 0.05, **q < 0.01. JDM-HQ (*n* = 14): **p < 0.01. JDM overall and JDM-HQ are higher than NOMID and HC; JDM overall is lower than CANDLE and SAVI. Abbreviations: IRG, interferon-regulated gene; JDM, juvenile dermatomyositis; JDM-HQ, highest-quartile scores in JDM; CANDLE, Chronic Atypical Neutrophilic Dermatosis with Lipodystrophy and Elevated temperature; SAVI, STING-Associated Vasculopathy with onset during Infancy; NOMID, neonatal-onset multisystem inflammatory disease; HC, healthy controls; IQR, interquartile range

JDM patients (median 40) had higher 28 IRG-S than patients with NOMID and HC and lower IRG-S than CANDLE and SAVI patients (median 534 and 501, respectively). IRG-S of JDM-HQ patients (median 473) did not differ from CANDLE and SAVI patients (Fig. 1). The IRG-S for each of the MSA groups were higher than in patients with NOMID and HC and did not differ from each other. IRG-S of JDM patients with anti-TIF1 and anti-NXP2 autoantibodies (median 42 and 25, respectively) were lower than those of patients with CANDLE and SAVI. Anti-MDA5 autoantibody-positive and MSA-negative patients (median 147 and 186, respectively) are lower but not statistically different from CANDLE and SAVI (Additional file 1: Figure S1).

### Principal component analysis (PCA)

**Fig. 2:**
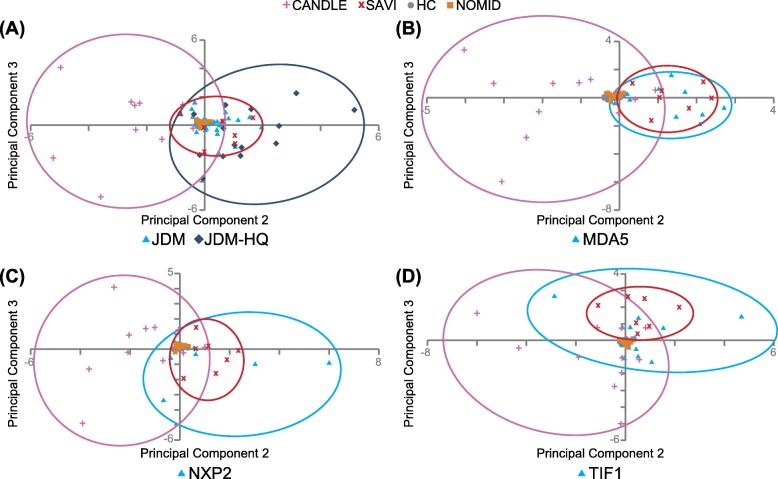
Principal component 2 (PC2) and 3 (PC3) scores from principal component analysis (PCA) of interferon-regulated gene (IRG) scores from JDM patients with other conditions. **a** PCA-A with JDM (including JDM highest quartile IRG score, JDM-HQ), CANDLE, SAVI, NOMID, and HC. **b** PCA-B with anti-MDA5 autoantibody-positive subgroup of JDM, CANDLE, SAVI, NOMID, and HC. **c** PCA-C with anti-NXP2 autoantibody-positive subgroup of JDM, CANDLE, SAVI, NOMID, and HC. **d** PCA-D with anti-TIF1 autoantibody-positive subgroup of JDM, CANDLE, SAVI, NOMID, and HC. Dotted circles represent each group, including CANDLE (pink), SAVI (red), and JDM-HQ or JDM myositis-specific autoantibody (MSA) group (anti-TIF1, anti-NXP2, anti-MDA5) (blue). JDM, as opposed to JDM-HQ, in **a** represents the lower 3 quartiles of JDM IRG scores. Abbreviations: IRG, interferon-regulated gene; JDM, juvenile dermatomyositis; JDM-HQ, highest-quartile scores in JDM; TIF1, subgroup of JDM with anti-TIF1 myositis-specific autoantibodies; NXP2, subgroup of JDM with anti-NXP2 myositis-specific autoantibodies; MDA5, subgroup of JDM with anti-MDA5 myositis-specific autoantibodies; neg, subgroup of JDM that was negative on testing for myositis-specific autoantibodies; CANDLE, Chronic Atypical Neutrophilic Dermatosis with Lipodystrophy and Elevated temperature; SAVI, STING-Associated Vasculopathy with onset during Infancy; NOMID, neonatal-onset multisystem inflammatory disease; HC, healthy controls

All 5 PCA analyses had three interpretable principal components (PCs) with eigenvalues > 1, accounting for 89.3–91.3% of the variance. All 5 PCAs produced a similar PC1 with high (> 0.8) component loadings for 23–25 out of 28 genes accounting for 80.4–82.8% of overall variance and did not distinguish CANDLE, SAVI, and JDM well (Additional file 1: Table S3). Thus, PC1 differentiates IFN disease groups (CANDLE, SAVI, JDM, and JDM MSA groups) from IFN-negative controls (NOMID and HC). PC2 and PC3 better demonstrated the differentiation among CANDLE, SAVI, and JDM overall (PCA-A, Fig. 2a), or the specific MSA groups within JDM from CANDLE and SAVI (PCA-B to PCA-D, Fig. 2b–d; PCA-E, Additional file 1: Figure S2). CANDLE, SAVI, and JDM or the specific MSA group separated from HC and NOMID in PC2 and PC3. JDM-HQ patients are further separated from HC and NOMID than the remaining JDM patients. In PC2 and PC3, JDM overlaps more with SAVI than CANDLE. Anti-MDA5 autoantibody-positive JDM patients show near-complete overlap with SAVI (PCA-B, Fig. 2b), in contrast to anti-NXP2 and anti-TIF1 autoantibody-positive JDM, and the MSA-negative groups, which overlap more with SAVI than CANDLE, but to a lesser degree (PCA-C, PCA-D, Fig. 2c, d; PCA-E, Additional file 1: Figure S2).

Across all five PCAs, we identified the genes with the largest component loadings in PC2 and PC3 (absolute value > 0.4). Briefly, component loadings are essentially weights representing the relative contributions of each gene to the principal component (linear combinations of individual gene scores). In 4 of the 5 PCAs (for JDM overall and all subgroups except anti-TIF1), PC2 had large loadings for *GBP1* and *SOCS1*, and PC3 had large loadings for *CXCL10* (Table 3). *IFI27* was a large contributor to PC2 in PCAs that included JDM overall, as well as anti-TIF1 and anti-NXP2 autoantibody-positive patients, and a large PC3 contributor for PCAs that included anti-MDA5 autoantibody-positive and MSA-negative patients. *LAMP3* had a large PC2 loading only for the PCA that included anti-MDA5 autoantibody-positive patients. The anti-TIF1 autoantibody-positive PCA had *CXCL10* in PC2 and *GBP1* and *SOCS1* in PC3.

**Table 3:** Highest magnitude component loadings from PCA (PC2, PC3) for JDM/MSA subgroups versus other conditions

Gene	PCA-A with JDM		PCA-B with anti-MDA5 Ab		PCA-C with anti-NXP2 Ab		PCA-D with anti-TIF1 Ab		PCA-E with MSA-neg	
	PC2	PC3	PC2	PC3	PC2	PC3	PC2	PC3	PC2	PC3
*CXCL10*	− 0.102	**− 0.667**	*− 0.267*	**− 0.666**	*−0.321*	**− 0.685**	**− 0.659**	*0.336*	*− 0.259*	**− 0.686**
*GBP1*	**− 0.506**	*0.215*	**− 0.562**	0.167	**− 0.448**	*0.216*	*− 0.200*	**− 0.473**	**0.517**	− 0.076
*IFI27*	**− 0.510**	*− 0.345*	*− 0.218*	**− 0.467**	− **0.444**	*− 0.250*	**− 0.579**	0.062	0.145	**− 0.546**
*LAMP3*	*− 0.216*	0.059	**− 0.444**	0.049	*− 0.325*	− 0.021	− 0.066	*− 0.311*	*0.349*	− 0.177
*SIGLEC1*	0.132	**− 0.424**	*0.338*	*− 0.330*	0.017	*− 0.348*	*− 0.204*	*0.315*	*− 0.343*	*− 0.224*
*SOCS1*	**− 0.428**	*0.300*	**− 0.437**	0.155	**− 0.413**	*0.323*	− 0.085	**− 0.503**	**0.486**	0.061

Bold indicates an absolute value of component loading > 0.4 (high magnitude)

Italic and underline indicates an absolute value of component loading between 0.2 to 0.4

PCA-A: PCA of JDM, CANDLE, SAVI, NOMID, and HC

PCA-B: PCA of Anti-MDA5 Ab, CANDLE, SAVI, NOMID, and HC

PCA-C: PCA of Anti-NXP2 Ab, CANDLE, SAVI, NOMID, and HC

PCA-D: PCA of Anti-TIF1 Ab, CANDLE, SAVI, NOMID, and HC

PCA-E: PCA of MSA-neg, CANDLE, SAVI, NOMID, and HC

The component loadings are the correlations of the individual gene normalized *Z*-scores with the given principal component. A stronger loading (e.g., greater than 0.40 or less than − 0.40) indicates a stronger relationship or contribution of that gene with that principal component. As with correlations, these can be negative or positive. The highest magnitude component loading interferon-regulated genes are listed on the left. The sign or direction of the component loading is arbitrary and can be reversed with the same interpretation, so the focus should be on the weight or magnitude

Abbreviations: *PCA* principal component analysis, *PC* principal component, *JDM* includes all juvenile dermatomyositis patients, *MSA* myositis-specific autoantibody group, *anti-TIF1 Ab* includes subgroup of JDM patients with anti-TIF1 autoantibodies, *anti-NXP2 Ab* subgroup of JDM patients with anti-NXP2 autoantibodies, anti*-MDA5 Ab* includes subgroup of JDM patients with anti-MDA5 autoantibodies, *MSA-neg* includes subgroup of JDM that is negative for myositis-specific autoantibodies, *CANDLE* Chronic Atypical Neutrophilic Dermatosis with Lipodystrophy and Elevated temperature, *SAVI* STING-Associated Vasculopathy with onset during Infancy, *NOMID* neonatal-onset multisystem inflammatory disease

### Gene proportion analysis

**Fig. 3:**
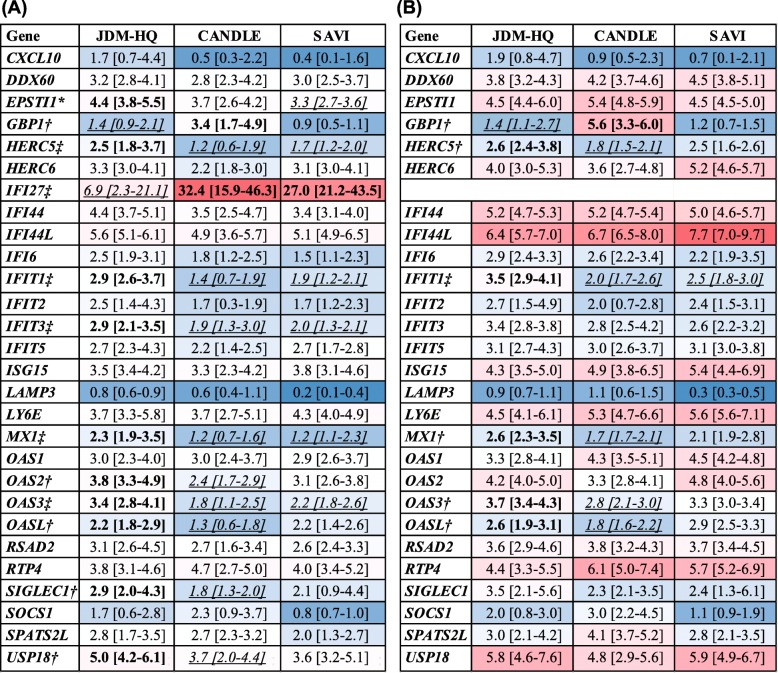
Proportion of individual genes from 28 normalized IRG-S for JDM highest quartile (JDM-HQ), CANDLE, SAVI. *JDM-HQ gene proportion significantly different from SAVI. ^†^JDM-HQ gene proportion significantly different from CANDLE. ^‡^JDM-HQ gene proportion significantly different from CANDLE and SAVI. Heat map of median proportion of the respective gene by normalized *Z*-score with red for highest proportion and dark blue for lowest proportion. Bold indicates values in a diagnosis group(s) significantly higher compared to values in the italicized/underlined group(s). Italicized/underlined indicates values in a diagnosis group(s) significantly lower compared to values in bold group(s). **a** All 28 IRGs are included. **b** IFI27 has been removed. Individual gene percentages (%) of the total IRG score, by normalized *Z*-scores (see the “Methods” section) are shown in alphabetical order by gene as median and interquartile ranges for JDM highest-quartile, CANDLE, and SAVI patients. Medians percentages of each gene by diagnosis may not sum to 100 due to skewing in the group, but they do in individual patients. JDM-HQ (*n* = 14) included 4 with anti-TIF1 Ab, 4 with anti-MDA4 Ab, 3 with anti-NXP2 Ab, and 3 MSA-neg. The JDM Physician Global Activity (PGA) median is 4.1/10 with an interquartile range of 2.4 to 4.7. Abbreviations: IRG, interferon-regulated gene; JDM-HQ, juvenile dermatomyositis patients with the highest quartile of IRG scores; CANDLE, Chronic Atypical Neutrophilic Dermatosis with Lipodystrophy and Elevated temperature; SAVI, STING associated Vasculopathy with onset during Infancy; anti-TIF1 Ab, includes subgroup of JDM patients with anti-TIF1 autoantibodies; anti-NXP2 Ab, subgroup of JDM patients with anti-NXP2 autoantibodies; anti-MDA5 Ab, includes subgroup of JDM patients with anti-MDA5 autoantibodies; MSA-neg, includes subgroup of JDM that is negative for myositis-specific autoantibodies; PGA. Physician Global Activity on 0–10 visual analog scale

The contribution of individual genes to the normalized total IRG-S was evaluated among CANDLE, SAVI, and JDM-HQ (*n* = 14). JDM-HQ was chosen for comparison as overall, it has IRG-S in the same range as CANDLE and SAVI in order to compare gene proportions more equitably. *IFI27* constituted a significantly smaller proportion of the 28-gene IRG-S in the JDM-HQ (median 6.9%) than in CANDLE (median 32.4%) or SAVI (median 27.0%) (Fig. 3a). Because *IFI27* dominated the IRG-S in CANDLE and SAVI and the proportion of total IRG-S was higher in JDM-HQ for many other IRGs, analysis comparing contribution of other genes to the overall score was done after removing *IFI27*. Without *IFI27*, only *IFIT1* remained significantly greater in proportion in JDM-HQ versus SAVI and CANDLE. *GBP1* continued to constitute a greater percentage of the IRG-S in CANDLE than JDM-HQ, while four IRGs (*HERC5*, *MX1*, *OAS3*, *OASL)* had significantly greater proportion in JDM-HQ than in CANDLE (Fig. 3b).

### Ratios related to NF-κB and IFN-γ

The NF-κB ratio in the JDM patients with elevated IRG-S (median 0.03) was significantly lower than NOMID (median 0.13) and HC (median 0.10). They were in the range of the CANDLE (median 0.06) and SAVI (median 0.02) patients, consistent with a smaller cohort of JDM patients previously assessed (Additional file 1: Figure S3A) [26]. When examining JDM patients by the MSA group, the anti-MDA5 group (median 0.02) has the lowest ratio (Additional file 1: Figure S3B). Regarding the IFN-γ ratio, the JDM patients with elevated IRG-S have a significantly higher ratio (median 0.44) versus CANDLE (median 0.38) and also have a higher ratio than SAVI (median 0.41, *p* = 0.06) (Additional file 1: Figure S4A). When examining JDM patients by MSA groups, the anti-MDA5 group (median 0.44) has the lowest ratio (Additional file 1: Figure S4B).

### IRG score correlations in JDM

The standard IRG-S correlated moderately (*r*_*s*_ = 0.33–0.47) with seven measures of disease activity in JDM, including physician global activity (PGA), manual muscle testing (MMT), extra-muscular global and skeletal activity, Disease Activity Score (DAS), and 2 serum muscle enzyme levels, with similar performance 2 published IRG-S (Table 2). In anti-TIF1 autoantibody-positive JDM patients, IRG-S more strongly correlated with cutaneous and extra-muscular activity, as well as DAS total and skin activity (*r*_*s*_ = 0.58–0.76), with lower correlation with MMT. Other IRG-S performed similarly for the anti-TIF1 autoantibody-positive JDM group (data not shown). Limited patient assessments precluded correlation of IRG-S with disease measures in anti-NXP2 and anti-MDA5 autoantibody-positive patients, although there was no difference in PGA among MSA subgroups.

Forty-six percent (26/57) of JDM patients had high IRG-S (> 48.9). JDM patients with high IRG-S (> 48.9) differed in 11 disease activity measures from those with low scores (Additional file 1: Table S1). From the multivariable regression analysis, low muscle strength (odds ratio [OR] 0.91, *p* < 0.01) and high skeletal (joint) disease activity (OR 3.3, *p* = 0.03) were the best predictors of high IRG-S (Table 4). No significant differences in high versus low IRG-S were found by gender, race, disease duration, or ever having calcinosis, cutaneous ulceration, or interstitial lung disease (Additional file 1: Table S1). Unfortunately, small numbers within specific MSA groups precluded multivariable regression analysis within a MSA group.

**Table 4:** Multivariable logistic regression predicting high IFN score in JDM patients

Parameter	Estimate	Standard error	*p* value	Odds ratio	95% confidence interval	
					Lower	Upper
Constant	23.247	8.968	**0.010**	–	–	–
Total MMT score	− 0.099	0.037	**0.007**	0.91	0.84	0.97
LDH	− 0.013	0.007	0.073	0.99	0.97	1.00
Skeletal VAS	1.184	0.550	**0.031**	3.27	1.11	9.60

High and low 28 interferon-regulated gene (IRG) score is defined by above and below 48.9, 95th percentile of healthy controls [22]. Backwards stepwise regression was performed (alpha < 0.15). *p* values < 0.05 are bolded. With the odds ratio, 1 is 1 point in the MMT score, LDH value, or 1 point on 10 point scale of skeletal VAS

Abbreviations: *Total MMT score* 26 muscle manual muscle testing (0–260), *LDH* lactate dehydrogenase, *skeletal VAS* Myositis Disease Activity Assessment Tool Skeletal disease activity visual analog scale

Regarding therapy (Additional file 1: Table S4), neither oral nor intravenous corticosteroid doses correlated with IRG-S (*r*_*s*_ = 0.04–0.08). No differences in IRG-S were evident among patients grouped by total steroid-sparing medications received. Patients receiving hydroxychloroquine (*n* = 21/56), methotrexate (*n* = 40/56), or intravenous immunoglobulin (*n* = 18/56) had no difference in IRG-S compared to those not receiving these therapies. Patients receiving mycophenolate mofetil (MMF) (*n* = 8/56) had lower IRG-S than those not receiving MMF (median − 4.29 vs. 85.2, *p* = 0.004), although they also had significantly lower PGA (median 1.7 vs. 2.3, *p* = 0.03).

Given the increased overlap of the anti-MDA5 subgroup of the JDM cohort (*n* = 10 with clinical information), we assessed the clinical characteristics of this group in particular versus the rest of JDM, CANDLE, and SAVI (Additional file 1: Table S2). The anti-MDA5 subgroup of JDM versus the rest of JDM was characterized by increased cutaneous ulceration (7/10, 70% vs. 8/46, 17%) and interstitial lung disease (ILD) (7/10, 70% vs. 1/46, 2%) with less weakness defined as a MMT score of < 225/260 (0/8, 0.0% vs. 8/28, 29%). In our SAVI cohort, distal ulcerations (4/4, 100.0%) and ILD (5/5, 100.0%) are common, while both are not features commonly observed in our CANDLE cohort (none reported and 3/9, 33.3%, respectively). Myositis is not a frequently observed or assessed feature in our SAVI patients (1/4, 25.0%) and more common in our CANDLE cohort (8/10, 80.0%) though MMT was not performed. Overall, there are multiple features overlapping between JDM, CANDLE, and SAVI with the highest overlap between anti-MDA5 autoantibody-positive JDM patients with SAVI.

To compare the interferon-regulated gene expression to protein expression, the IRG-S was compared to IP-10 in JDM (*n* = 19). A significant correlation was found (*r*_*s*_ = 0.49, *p* = 0.03), similar to that observed in CANDLE and SAVI patients [20].

## Discussion

This study analyzed an IRG-S in JDM and MSA groups within JDM versus Mendelian interferonopathies (CANDLE and SAVI), an IL-1 mediated, non-IFN autoinflammatory disease control (NOMID), and healthy controls. PCA and gene proportion analysis suggested similarities of the IRG pattern elevation in JDM and anti-MDA5 autoantibody-positive patients to SAVI and a moderate correlation of IRG-S with clinical measures.

Blood IRG-S in JDM patients ranged from the levels in HC to as high as CANDLE, and SAVI, but were primarily between HC/NOMID and CANDLE/SAVI. We confirmed that some JDM patients’ IRG-S overlap with HC (54% in our cohort) [7, 28]. *IFI27* was found to be most dynamically upregulated [22], contributing a higher proportion when the IRG-S is higher (data not shown). We found that although *IFI27* contributed the most to the total IRG-S in JDM-HQ, SAVI, and CANDLE with similarly high overall scores, the proportion of *IFI27* to the total IRG-S was lower in JDM-HQ. After excluding *IFI27*, four genes (*HERC5*, *MX1*, *OAS3*, *OASL*) contributed a higher proportion to the total IRG-S in JDM-HQ versus CANDLE, whereas one gene, *IFIT1*, had an increased proportion in JDM-HQ compared to both CANDLE and SAVI. IFIT1 is a negative regulator of STING, which is an adaptor protein essential for IFN-β induction. IFIT1 disrupts the STING interaction with mitochondrial anti-viral signaling protein (MAVS) and TANK-binding kinase (TBK1), which decreases IFN-β expression [29], and may present a counter-regulatory mechanism to “dampen” STING and IFN-β signaling in JDM. Interestingly, SAVI, caused by gain-of-function mutations in STING leading to constitutive activation of IFN-β through the STING-TBK1-IRF3 pathway [16], has a more complete overlap of IRG-S with JDM than CANDLE suggesting that STING and IFN-β may be preferentially activated in JDM. In DM, the higher correlation of serum IFN-β with disease activity and peripheral blood IRG signature versus IFN-α [10] could be consistent with a pathogenic role of STING in JDM. JDM patients with elevated IRG-S had lower NF-κB ratios, similar to canonical interferonopathies CANDLE and SAVI, indicating it is unlikely JDM has much concomitant NF-kB signaling seen in other conditions with an elevated IRG-S [26]. Overall, JDM with elevated IRG-S had higher IFN-γ ratios than CANDLE and SAVI, which is consistent with previous reports of IFN-γ co-localizes with CD3^+^ T cells in untreated JDM muscle biopsies and increase in both type I and type I interferon-regulated genes in JDM muscle [8], which may differentiate JDM from autoinflammatory CANDLE and SAVI. Differences in these ratios by MSA groups merit evaluation in larger groups.

SAVI is a vasculopathy characterized by violaceous plaques and/or nodules, often with features of vascular damage with gangrene and infarcts in acral areas such as at the tips of the fingers and/or toes [16, 17]. JDM is also a small-vessel vasculopathy with dilated and tortuous periungual capillaries, endarteropathy, and capillary loss noted on muscle biopsy, and patients may develop vasomotor instability in acral areas [3, 30, 31]. Both JDM and SAVI share features of vasculopathy leading to thrombosis, tissue ischemia, and infarction, consistent with both phenotypic overlap and potentially shared role of IFN in vasculopathy pathogenesis [16, 17, 30, 31]. Although Janus kinase (JAK) inhibitor therapy does not specifically or fully block IFN-β or constitutively activated STING, it does inhibit type I and II IFN signaling [32] and several reports have noted clinical improvement in SAVI with decreased skin vasculopathy and stabilization of lung disease on JAK inhibitor therapy [20, 33]. Similarly, a limited number of cases in refractory JDM patients have noted improvement of skin and muscle symptoms with JAK inhibitor therapy [34, 35, 38].

MSA groups continue to evolve as an important predictor of subgroups within JDM both regarding phenotype and prognosis. Among the IRG-S of MSA group patients, the anti-MDA5 autoantibody-positive IRG-S overlapped most closely with SAVI by PCA. The anti-MDA5 autoantibody subgroup of JDM is characterized by increased cutaneous ulceration and interstitial lung disease (ILD) with less muscle disease [1–3], also observed in our cohort, with more overlap with these vasculopathic features seen in SAVI than CANDLE in general [15–17], also specifically observed in our cohort. JAK inhibitor therapy has been noted to show improved outcomes in refractory anti-MDA5 autoantibody adult and juvenile dermatomyositis, including with rapidly progressive ILD [36–38].

Furthermore, the peripheral blood IRG-S correlates moderately with disease activity measures, including muscle and extra-muscular components [6, 7, 10]. Multivariable regression analysis identified weakness (MMT-26) and increased skeletal features (skeletal VAS) as the most important factors associated with high IRG-S in JDM overall. MSA-negative patients having elevated IRG-S differs from previous reports of lower IFN chemokine scores in MSA-negative patients, although this was a different type of score and the methodology to determine MSAs also differed [39]. In anti-TIF1 autoantibody-positive patients, who characteristically tend to have more photosensitive rashes, the IRG-S correlated more strongly with skin disease-activity measures although anti-TIF1 patients did not have significantly higher skin activity in our cohort. Unfortunately, small numbers of patients precluded multivariable regression analysis within the anti-TIF1 MSA group.

Though type I IFN itself is in extremely low concentrations in the blood or serum, IRGs are readily quantifiable and have been associated with elevation of serum IFN-α and IFN-β in DM [7, 10]. Notably, our gene expression assessment on NanoString™, which does not require amplification, has low inter-assay and inter-observer variability and excellent reproducibility [22] and may hold value as a potential biomarker in the clinical setting compared to a traditional polymerase chain reaction or PCR-based methods used in other studies [6, 7, 28] that are more difficult to standardize particularly across centers. Additionally, the IRG-S correlated significantly with peripheral protein IP-10. The “gold standard” to validate the peripheral IFN score would be a comparison to the affected skin and/or muscle, in which elevated IRG expression has been previously reported [8, 9]. Although correlations between skin/muscle and blood IRG expression have not been performed, the correlation of the blood IRG expression with muscle and skin disease activity in our Nanostring™ assay and other reports using PCR analyses [6, 7, 10] holds promise as a valuable disease activity marker, though further validation is needed.

Limitations of this study include the evaluation of referred JDM patients at a single time point who have received multiple immunosuppressive therapies. We generally did not find that the IRG-S correlated with any specific medications. However, muscle IRG expression in DM and adult polymyositis and peripheral interferon-regulated chemokine score in refractory adult and pediatric myositis was reported to be reduced with rituximab therapy [40, 41]. The number of patients with Mendelian autoinflammatory diseases and within MSA subgroups was small, but the uniform elevation of the IRG signature and disease-specific differences still allowed for meaningful analysis. Lastly, we evaluated whole blood; thus, the differential contributions of various cell types to the IRG elevation could not be assessed. Though we could not normalize for specific different cell populations within whole blood, it is reassuring that exploratory analysis of white blood cells and differential cell counts between JDM versus CANDLE and SAVI did not find any significant differences (data not shown).

## Conclusions

In conclusion, the IRG-S correlates with disease measures in JDM, with a higher correlation for cutaneous features in the anti-TIF1 autoantibody group. IRG-S in JDM has similarities to that of Mendelian interferonopathies, particularly IRG-S for the JDM-HQ and anti-MDA5 autoantibody subgroups. Further evaluation by the MSA group in larger cohorts is important to examine. Specific IRGs have a differential contribution in JDM patients, with a pattern more similar to SAVI than CANDLE, pointing to a potentially larger role of STING activation and IFN-β in JDM and a potential treatment target.

## Supplementary information

**Additional file 1: Table S1.** Univariate Comparison of Parameters in JDM patients with High versus Low Interferon-Regulated Gene Score. **Table S2.** Clinical Features of JDM and myositis-specific autoantibody (MSA) groups, CANDLE, and SAVI. **Table S3.** Component loadings for Principal Component (PC) Analysis for PC1, PC2 and PC3 for JDM or myositis-specific autoantibody (MSA) subgroups of JDM, with autoinflammatory conditions and controls. **Table S4.** Treatment information. **Figure S1.** Peripheral blood interferon-regulated gene (IRG) scores in juvenile dermatomyositis (JDM) myositis-specific autoantibody (MSA) groups compared to monogenic autoinflammatory disease patients and healthy controls. **Figure S2.** PCA graph of Principal Component 2 and 3 scores with MSA-negative JDM group with other conditions. **Figure S3.** NF-κB ratio in JDM with elevated IRG-S and by MSA groups versus other conditions. **Figure S4.** IFNγ ratio in JDM with elevated IRG-S and by MSA groups versus other conditions.

## Data Availability

The datasets generated and analyzed during the current study are not publicly available due to their containing information that could compromise research participant privacy. The data are available and de-identified from the corresponding author (HK) on reasonable request.

## Abbreviations

JDM: Juvenile dermatomyositis
IFN: Interferon
CANDLE: Chronic Atypical Neutrophilic Dermatosis with Lipodystrophy and Elevated temperature
SAVI: STING-Associated Vasculopathy with onset in Infancy
IRG-S: Interferon-regulated gene score
MSA: Myositis-specific autoantibodies
HC: Healthy controls
PCA: Principal component analysis
IRG: Interferon-regulated genes
DM: Dermatomyositis
STING: STimulator of INterferon Genes
JAK: Janus kinase
NOMID: Neonatal-onset multisystem inflammatory disease
NIH: National Institutes of Health
NIEHS: National Institute of Environmental Health Sciences
IRB: Institutional Review Board
NIAID: National Institute of Allergy and Infectious Diseases
JDM-HQ: Highest quartile of juvenile dermatomyositis interferon-regulated gene scores
MDAAT: Myositis Disease Activity Assessment Tool
VAS: Visual analog scale
PC: Principal component
PGA: Physician global activity assessment by visual analog scale
MMT: Manual muscle testing
DAS: Disease Activity Score
MMF: Mycophenolate mofetil
ILD: Interstitial lung disease
MAVS: Mitochondrial anti-viral-signaling protein
TBK1: TANK-binding kinase
PCR: Polymerase chain reaction
IP-10: Interferon gamma-inducible protein 10
NF-κB: Nuclear factor kappa B
