# Supplementary material for: Expression of interferon-regulated genes in juvenile dermatomyositis versus Mendelian autoinflammatory interferonopathies
Source: Arthritis Res Ther. 2020 Apr 6;22:69. doi: 10.1186/s13075-020-02160-9 (PMC7137415; doi:10.1186/s13075-020-02160-9)
Supplement: Supplementary file 1 — Additional file 1: Table S1. Univariate Comparison of Parameters in JDM patients with High versus Low Interferon-Regulated Gene Score. Table S2. Clinical Features of JDM and myositis-specific autoantibody (MSA) groups, CANDLE, and SAVI. Table S3. Component loadings for Principal Component (PC) Analysis for PC1, PC2 and PC3 for JDM or myositis-specific autoantibody (MSA) subgroups of JDM, with autoinflammatory conditions and controls. Table S4. Treatment information. Figure S1. Peripheral blood interferon-regulated gene (IRG) scores in juvenile dermatomyositis (JDM) myositis-specific autoantibody (MSA) groups compared to monogenic autoinflammatory disease patients and healthy controls. Figure S2. PCA graph of Principal Component 2 and 3 scores with MSA-negative JDM group with other conditions. Figure S3. NF-κB ratio in JDM with elevated IRG-S and by MSA groups versus other conditions. Figure S4. IFNγ ratio in JDM with elevated IRG-S and by MSA groups versus other conditions. [file 13075_2020_2160_MOESM1_ESM.docx]

**Supplemental Table S1.** Univariate Comparison of Parameters in JDM patients with High versus Low Interferon-Regulated Gene Score.

|  | **Median [IQR] (n)** | | |  | |
| --- | --- | --- | --- | --- | --- |
| **Disease measure**  **(units, range, upper limit  of normal (ULN))** | **High IRG score (n=26)** | **Low IRG score (n=31)** | **p-value** | |  |
| Age (years) | 9.9 [5.4 - 12.9] (n=26) | 9.1 [6.3 - 13.3] (n=31) | 0.921 | |  |
| Disease Duration (years) | 0.7 [0.4 - 2.6] (n=25) | 1.1 [0.5 – 5.3] (n=31) | 0.190 | |  |
| Number of therapies* | 1.0 [0.5-2.0] (n=25) | 1.0 [1.0-2.0] (n=31) | 0.070 | |  |
| Physician Global Activity (0-10 VAS)† | 3.2 [2.3 - 4.5] (n=25) | 1.8 [1.4 - 2.5] (n=31) | **<0.001** | |  |
| MMT8 (0-80) | 68 [59 - 73] (n=13) | 73 [70 - 79] (n=23) | **0.012** | |  |
| MMT26 (0-260)† | 226 [200 - 243] (n=13) | 248 [235 - 257] (n=23) | **0.002** | |  |
| CMAS (0-52) | 39 [32 - 48] (n=14) | 46 [39 - 48] (n=24) | 0.103 | |  |
| CHAQ /HAQ(0-3.000) | 1.06 [0.375 - 1.56] (n=22) | 0.5 [0.125 – 1.000] (n=20) | 0.067 | |  |
| MDAAT (0-10 VAS) |  |  |  | |  |
| Muscle | 2.8 [2.1 – 4.0] (n=15) | 1.4 [0.8 – 2.1] (n=24) | **0.004** | |  |
| Constitutional | 1.3 [0.3 – 4.0] (n=15) | 1.3 [0.3 - 2.9] (n=24) | 0.442 | |  |
| Cutaneous† | 3.8 [2.0 – 5.0] (n=15) | 2.2 [0.9 - 2.8] (n=24) | **0.008** | |  |
| Skeletal† | 1.7 [0.9 – 4.1] (n=15) | 0.5 [0.0 – 1.8] (n=24) | **0.014** | |  |
| Pulmonary | 0.7 [0.3 – 2.5] (n=15) | 0.2 [0.0 – 1.4] (n=24) | 0.136 | |  |
| Extramuscular Activity (0-10 VAS) | 3.5 [1.7 – 4.6] (n=16) | 1.6 [1.0 – 2.0] (n=24) | **0.002** | |  |
| Disease Activity Score Total (0-20) | 13 [11 – 14] (n=15) | 10 [9 – 13] (n=24) | **0.010** | |  |
| DAS Skin (0-9) | 7 [5 – 8] (n=16) | 5 [5 – 6] (n=24) | 0.054 | |  |
| DAS Muscle (0-11) | 6 [4- 8] (n=15) | 5 [3 – 6] (n=24) | 0.089 | |  |
| Serum Muscle Enzymes (U/L) |  |  |  | |  |
| Aldolase (ULN 252 U/L)† | 8.6 [5.8- 10.7] (n=24) | 6.5 [4.9 – 8.0] (n=30) | **0.023** | |  |
| AST (ULN 34 U/L)† | 30 [22 – 37] (n=25) | 20 [16 – 27] (n=31) | **0.001** | |  |
| CK (ULN 252 U/L) | 83 [51 – 145] (n=25) | 100 [64 – 196] (n=31) | 0.323 | |  |
| LDH (ULN 226 U/L)† | 211 [179 – 254] (n=25) | 168 [151 – 198] (n=31) | **<0.001** | |  |
| Physician Global Damage (0-10 VAS) | 0.9 [0.3 – 1.7] (n=22) | 1.1 [0.5 – 2] (n=24) | 0.502 | |  |
| MDI Total Severity (0-110) | 5 [2 – 9] (n=11) | 3 [1 – 7] (n=23) | 0.364 | |  |
|  | **(n/total, %)** | |  | |  |
| Female | 17/26 (65%) | 17/31 (55%) | 0.588 | |  |
| White | 14/26 (54%) | 22/31 (71%) | 0.271 | |  |
| Calcinosis | 4/21 (19%) | 3/24 (13%) | 0.689 | |  |
| Cutaneous Ulceration | 5/16 (31%) | 3/23 (13%) | 0.235 | |  |
| Interstitial Lung Disease | 4/22 (18%) | 3/24 (13%) | 0.694 | |  |

* Includes all immunosuppressive drugs and biologics, except corticosteroid therapy.

† Included in logistic regression analysis.

High and low 28 interferon-regulated gene (IRG) score is defined by above and below 48.9 (95th percentile of healthy controls) (22). P values <0.05 are **bolded**.

Abbreviations: IRG: Interferon-regulated gene score; JDM: juvenile dermatomyositis; VAS: visual analog scale; MMT: manual muscle testing; CMAS: Childhood Myositis Assessment Scale; CHAQ: Childhood Health Assessment Questionnaire; HAQ: Health Assessment Questionnaire, MDAAT: Myositis Disease Activity Assessment Tool; DAS: Disease Activity Score; CK: creatine kinase; AST: aspartate aminotransferase; LDH: lactate dehydrogenase; PGD: Physician Global Damage; MDI: Myositis Damage Index.

**Supplemental Table S2.** Clinical Features of JDM and myositis-specific autoantibody (MSA) groups, CANDLE, and SAVI.

|  | **JDM Overall** | **TIF1 Ab JDM** | **NXP2 Ab JDM** | **MDA5 Ab JDM** | **Rest of JDM** | **CANDLE** | **SAVI** |
| --- | --- | --- | --- | --- | --- | --- | --- |
|  | **n=56** | **n=20** | **n=11** | **n=10** | **n=15** | **n=10** | **n=5** |
| **MMT score <225/260** | 8/36 (22.2%) | 3/13 (23.1%) | 2/5 (40.0%) | 0/8 (0.0%) | 3/10 (30.0%) | NA | NA |
| **Myositis*** |  |  |  |  |  | 8/10 (80.0%) | 1/4 (25%) |
| **Heliotrope** | 51/56 (91.1%) | 18/20 (90.0%) | 10/11 (90.9%) | 9/10 (90.0%) | 14/15 (93.3%) | None† | None† |
| **Gottron's papules** | 54/56 (96.4%) | 20/20 (100.0%) | 9/11 (81.8%) | 10/10 (100.0%) | 15/15 (100.0%) | None† | None† |
| **Photosensitivity** | 41/56 (73.2%) | 18/20 (90.0%) | 5/11 (45.5%) | 7/10 (70.0%) | 11/15 (73.3%) | None† | None† |
| **V-sign** | 24/56 (42.9%) | 11/20 (55.0%) | 1/11 (9.1%) | 5/10 (50.0%) | 7/15 (46.7%) | None† | None† |
| **Shawl sign** | 18/56 (32.1%) | 8/20 (55.0%) | 0/11 (0.0%) | 5/10 (50.0%) | 5/15 (33.3%) | None† | None† |
| **Panniculitis** | 5/45 (11.1%) | 1/20 (5.0%) | 1/11 (9.1%) | 1/10 (10.0%) | 2/15 (13.3%) | 4/4 (100.0%) | None† |
| **Nodular violaceous erythema and/or annular plaques** | None† | None† | None† | None† | None† | 4/4 (100.0%) | None† |
| **Cutaneous ulceration** | 15/56 (26.8%) | 4/20 (20.0%) | 3/11 (27.3%) | 7/10 (70.0%) | 1/15 (6.7%) | None† | 4/4 (100.0%) |
| **Interstitial lung disease** | 8/56 (14.3%) | 0/20 (0.0%) | 0/11 (0.0%) | 7/10 (70.0%) | 1/15 (6.7%) | 3/9 (33.3%) | 5/5 (100.0%) |
| **Fever** | 26/56 (46.4%) | 7/20 (35.0%) | 7/11 (63.6%) | 6/10 (60.0%) | 6/15 (40.0%) | 10/10 (100.0%) | 3/5 (60.0%) |
| **Adenopathy** | 19/56 (33.9%) | 5/20 (25.0%) | 5/11 (45.5%) | 5/10 (50.0%) | 4/15 (26.7%) | 5/10 (50.0%) | 3/4 (75.0%) |
| **Arthritis / arthralgia** | 46/56 (82.1%) | 16/20 (80.0%) | 10/11 (90.9%) | 10/10 (100.0%) | 10/15 (66.7%) | 8/9 (88.9%) | 1/5 (20.0%) |
| **Contractures** | 50/56 (89.3%) | 17/20 (85.0%) | 11/11 (100%) | 9/10 (90.0%) | 13/15 (86.7%) | 10/10 (100.0%) | 2/2 (100.0%) |
| **Calcinosis** | 15/56 (26.8%) | 5/20 (25.0%) | 3/11 (27.3%) | 4/10 (40.0%) | 3/15 (20.0%) | 3/10 (30.0%) | NA |
| **Lipodystrophy** | 8/56 (14.3%) | 6/20 (30.0%) | 2/11 (18.2%) | 0/10 (0.0%) | 0/15 (0.0%) | 10/10 (100.0%) | None† |

*: All the JDM patients have had proximal weakness.

†: This feature was not specifically documented in the medical records, but unlikely to have been present based on standard physical examinations performed.

Patients with clinical data available were classified for relevant clinical features. For some features, not all had data available.

Abbreviations: JDM: juvenile dermatomyositis; CANDLE: Chronic Atypical Neutrophilic Dermatosis with Lipodystrophy and Elevated temperature; SAVI: STING-Associated Vasculopathy with onset during Infancy; JDM Overall: includes all JDM patients; TIF1 Ab JDM: subgroup of JDM patients with anti-TIF1 autoantibodies; NXP2 Ab JDM: subgroup of JDM patients with anti-NXP2 autoantibodies; MDA5 Ab JDM: subgroup of JDM patients with anti-MDA5 autoantibodies; Rest of JDM: JDM patients without anti-TIF1, anti-NXP2, or anti-MDA5 autoantibodies.; MMT: manual muscle testing; NA: not assessed.

**Supplemental Table S3.** Component loadings for Principal Component (PC) Analysis for PC1, PC2 and PC3 for JDM or myositis-specific autoantibody (MSA) subgroups of JDM, with autoinflammatory conditions and controls.

| **Gene** | **PCA-A with JDM** | | | **PCA-B with  Anti-MDA5 Ab** | | | **PCA-C with  Anti-NXP2 Ab** | | | **PCA-D with  Anti-TIF1 Ab** | | | **PCA-E with MSA-neg** | | |
| --- | --- | --- | --- | --- | --- | --- | --- | --- | --- | --- | --- | --- | --- | --- | --- |
|  | **PC1** | **PC2** | **PC3** | **PC1** | **PC2** | **PC3** | **PC1** | **PC2** | **PC3** | **PC1** | **PC2** | **PC3** | **PC1** | **PC2** | **PC3** |
| *CXCL10* | 0.442 | -0.102 | -0.667 | 0.460 | -0.267 | -0.666 | 0.460 | -0.321 | -0.685 | 0.390 | -0.659 | 0.336 | 0.467 | -0.259 | -0.686 |
| *DDX60* | 0.977 | 0.020 | 0.033 | 0.980 | 0.036 | 0.004 | 0.979 | 0.024 | -0.010 | 0.979 | 0.019 | 0.031 | 0.972 | -0.034 | 0.046 |
| *EPSTI1* | 0.976 | -0.067 | 0.006 | 0.979 | -0.053 | 0.031 | 0.985 | -0.099 | 0.031 | 0.982 | -0.074 | -0.082 | 0.985 | 0.049 | -0.028 |
| *GBP1* | 0.756 | -0.506 | 0.215 | 0.780 | -0.562 | 0.167 | 0.808 | -0.448 | 0.216 | 0.822 | -0.200 | -0.473 | 0.808 | 0.517 | -0.076 |
| *HERC5* | 0.918 | 0.334 | 0.042 | 0.953 | 0.206 | 0.067 | 0.912 | 0.337 | -0.101 | 0.938 | 0.246 | 0.126 | 0.937 | -0.229 | 0.087 |
| *HERC6* | 0.922 | 0.121 | -0.182 | 0.911 | 0.287 | -0.152 | 0.898 | 0.109 | -0.168 | 0.913 | 0.041 | 0.327 | 0.905 | -0.307 | -0.024 |
| *IFI27* | 0.565 | -0.510 | -0.345 | 0.659 | -0.218 | -0.467 | 0.605 | -0.444 | -0.250 | 0.605 | -0.579 | 0.062 | 0.614 | 0.145 | -0.546 |
| *IFI44* | 0.982 | -0.008 | -0.011 | 0.984 | 0.010 | 0.002 | 0.981 | -0.023 | 0.049 | 0.977 | -0.028 | -0.029 | 0.982 | 0.038 | 0.020 |
| *IFI44L* | 0.976 | -0.058 | -0.043 | 0.984 | 0.049 | -0.024 | 0.981 | -0.050 | 0.040 | 0.981 | -0.051 | 0.025 | 0.985 | 0.017 | 0.017 |
| *IFI6* | 0.942 | -0.075 | -0.045 | 0.937 | -0.033 | -0.038 | 0.953 | -0.046 | 0.039 | 0.943 | -0.088 | -0.078 | 0.945 | 0.108 | -0.017 |
| *IFIT1* | 0.918 | 0.290 | -0.011 | 0.951 | 0.175 | 0.037 | 0.906 | 0.317 | -0.036 | 0.967 | 0.093 | 0.063 | 0.939 | -0.179 | 0.071 |
| *IFIT2* | 0.839 | 0.318 | 0.205 | 0.833 | 0.153 | 0.313 | 0.867 | 0.384 | 0.087 | 0.872 | 0.325 | 0.083 | 0.882 | -0.100 | 0.245 |
| *IFIT3* | 0.974 | 0.012 | 0.101 | 0.973 | -0.098 | 0.078 | 0.969 | 0.014 | 0.073 | 0.974 | 0.009 | -0.122 | 0.973 | 0.107 | 0.014 |
| *IFIT5* | 0.955 | 0.090 | 0.082 | 0.973 | 0.024 | 0.082 | 0.968 | 0.149 | 0.046 | 0.970 | 0.039 | 0.006 | 0.957 | -0.053 | 0.126 |
| *ISG15* | 0.903 | -0.152 | 0.016 | 0.921 | -0.036 | 0.048 | 0.907 | -0.085 | 0.115 | 0.914 | -0.042 | -0.020 | 0.893 | 0.059 | 0.018 |
| *LAMP3* | 0.808 | -0.216 | 0.059 | 0.791 | -0.444 | 0.049 | 0.779 | -0.325 | -0.021 | 0.826 | -0.066 | -0.311 | 0.795 | 0.349 | -0.177 |
| *LY6E* | 0.946 | -0.055 | 0.103 | 0.967 | 0.031 | 0.125 | 0.948 | -0.012 | 0.181 | 0.967 | 0.068 | -0.043 | 0.957 | 0.026 | 0.183 |
| *MX1* | 0.928 | 0.285 | -0.006 | 0.962 | 0.185 | -0.020 | 0.929 | 0.254 | -0.130 | 0.928 | 0.213 | 0.142 | 0.954 | -0.194 | 0.025 |
| *OAS1* | 0.963 | -0.085 | 0.044 | 0.969 | -0.038 | 0.076 | 0.971 | -0.038 | 0.100 | 0.966 | -0.026 | -0.054 | 0.976 | 0.049 | 0.086 |
| *OAS2* | 0.969 | 0.173 | -0.012 | 0.968 | 0.176 | 0.006 | 0.969 | 0.153 | -0.054 | 0.962 | 0.162 | 0.138 | 0.968 | -0.163 | 0.068 |
| *OAS3* | 0.962 | 0.188 | 0.040 | 0.979 | 0.117 | 0.025 | 0.974 | 0.143 | -0.033 | 0.956 | 0.199 | 0.046 | 0.976 | -0.101 | 0.075 |
| *OASL* | 0.953 | 0.121 | 0.114 | 0.947 | 0.111 | 0.135 | 0.950 | 0.161 | 0.091 | 0.949 | 0.185 | 0.043 | 0.946 | -0.087 | 0.184 |
| *RSAD2* | 0.981 | 0.033 | 0.087 | 0.987 | -0.051 | 0.102 | 0.990 | 0.036 | 0.079 | 0.986 | 0.084 | -0.056 | 0.985 | 0.037 | 0.055 |
| *RTP4* | 0.943 | -0.186 | 0.104 | 0.960 | -0.095 | 0.105 | 0.943 | -0.138 | 0.185 | 0.967 | 0.017 | -0.087 | 0.956 | 0.140 | 0.100 |
| *SIGLEC1* | 0.773 | 0.132 | -0.424 | 0.755 | 0.338 | -0.330 | 0.862 | 0.017 | -0.348 | 0.849 | -0.204 | 0.315 | 0.848 | -0.343 | -0.224 |
| *SOCS1* | 0.743 | -0.428 | 0.300 | 0.816 | -0.437 | 0.155 | 0.782 | -0.413 | 0.323 | 0.780 | -0.085 | -0.503 | 0.809 | 0.486 | 0.061 |
| *SPATS2L* | 0.911 | -0.247 | -0.112 | 0.930 | -0.141 | -0.239 | 0.891 | -0.344 | -0.084 | 0.925 | -0.258 | -0.037 | 0.911 | 0.168 | -0.248 |
| *USP18* | 0.929 | 0.162 | -0.143 | 0.926 | 0.230 | -0.154 | 0.933 | 0.213 | -0.143 | 0.944 | 0.007 | 0.241 | 0.938 | -0.177 | -0.053 |

Heat map of component loadings with dark blue for highest component loadings, and dark red for most negative component loadings.

PCA-A: PCA of JDM, CANDLE, SAVI, NOMID, and HC.

PCA-B: PCA of Anti-MDA5 Ab, CANDLE, SAVI, NOMID, and HC.

PCA-C: PCA of Anti-NXP2 Ab, CANDLE, SAVI, NOMID, and HC.

PCA-D: PCA of Anti-TIF1 Ab, CANDLE, SAVI, NOMID, and HC.

PCA-E: PCA of MSA-neg, CANDLE, SAVI, NOMID, and HC.

Interferon regulated genes are listed on the left. The component loadings default to all positive in PC1. The sign or direction of the component loading is arbitrary and can be reversed with the same interpretation, so focus should be on the weight or magnitude. Results of five principal component analyses are included.

Abbreviations: PCA: principal component analysis; PC: principal component; JDM: includes all juvenile dermatomyositis patients; anti-TIF1 Ab: includes subgroup of JDM patients with anti-TIF1 autoantibodies; anti-NXP2 Ab: subgroup of JDM patients with anti-NXP2 autoantibodies; anti-MDA5 Ab: includes subgroup of JDM patients with anti-MDA5 autoantibodies; MSA-neg: includes subgroup of JDM that is negative for myositis-specific autoantibodies; CANDLE: Chronic Atypical Neutrophilic Dermatosis with Lipodystrophy and Elevated temperature; SAVI: STING-Associated Vasculopathy with onset during Infancy; NOMID: Neonatal-Onset Multisystem Inflammatory Disease.

**Supplemental Table S4**: Treatment information

| **Treatment*** | **n** | **median (IQR) mg/kg/day** |
| --- | --- | --- |
| oral prednisone† | 42 | 0.22 [0.01-0.50] |
| oral + IV steroid dose‡ | 16 | 0.32 [0.06-0.92] |
| MTX | 40 |  |
| HCQ | 21 |  |
| IVIG | 18 |  |
| MMF | 8 |  |
| cyclosporine | 6 |  |
| DMARD(s) § | 58 (1.04/patient) |  |
| Biologic(s) ¶ | 19 (0.34/patient) |  |
| DMARD(s) & Biologics (s)** | 77 (1.38/patient) |  |

*: Treatment information was available on 56/57 JDM patients

†: Includes prednisone equivalent prednisolone

‡: Of the 42 on oral steroids, 16 were also on IV steroids

§: DMARD (disease-modifying anti-rheumatic drugs) include methotrexate, azathioprine (n=3), cyclosporine, cyclophosphamide (n=1), mycophenolate mofetil. Number represents total number of DMARDs taken by all patients.

¶: Biologics: includes intravenous immunoglobulin and rituximab (n=1, received 10 months prior to IFN score assessment with detectable B cells at that time). Number represents total number of biologics taken by all patients.

**: Sum of DMARD and Biologics as defined above (§ and ¶)

Abbreviations: MTX: methotrexate; HCQ: hydroxychloroquine; IVIG: intravenous immunoglobulin; MMF: mycophenolate mofetil; DMARD: disease modifying anti-rheumatic drugs.

**Supplemental Figures**

**Figure S1**. Peripheral blood interferon-regulated gene (IRG) scores in juvenile dermatomyositis (JDM) myositis-specific autoantibody (MSA) groups compared to monogenic autoinflammatory disease patients and healthy controls.

Median and interquartile ranges shown. Dotted horizontal line represents 95^th^ %ile of healthy controls (22). CANDLE (n=11), SAVI (n=7), NOMID (n=18), HC (n=26).

*: p<0.05, **: p<0.01.

JDM patients with anti-TIF1 (n=20), anti-NXP2 (n=11), anti-MDA5 (n=11) autoantibodies and MSA-negative (n=9) are higher than NOMID and HC. TIF1 and NXP2 are lower than CANDLE and SAVI. There are 6 other JDM patients (3 with anti-Mi2, 2 with anti-synthetase autoantibodies, and 1 with MSA status unknown) with IRG scores below the JDM-HQ (median -1.6, IQR -4.4 to 174.2) that were not included.

Abbreviations: IRG: interferon-regulated gene; JDM: juvenile dermatomyositis; TIF1: subgroup of JDM with anti-TIF1 myositis-specific autoantibodies; NXP2: subgroup of JDM with anti-NXP2 myositis-specific autoantibodies; MDA5: subgroup of JDM with anti-MDA5 myositis-specific autoantibodies; MSA: myositis-specific autoantibody; MSA-neg: subgroup of JDM that had negative testing for myositis-specific autoantibodies; CANDLE: Chronic Atypical Neutrophilic Dermatosis with Lipodystrophy and Elevated temperature; SAVI: STING-Associated Vasculopathy with onset during Infancy; NOMID: Neonatal-Onset Multisystem Inflammatory Disease; HC: healthy controls; IQR: interquartile range.

**Figure S2**. PCA graph of Principal Component 2 and 3 scores with MSA-negative JDM group with other conditions.

Graph of PCA-E with MSA-negative subgroup of JDM, CANDLE, SAVI, NOMID, and HC. Large circles represent the individuals within each group, including CANDLE (pink), SAVI (red), and MSA-neg (blue).

Abbreviations: PCA: principal component analysis; JDM: juvenile dermatomyositis; CANDLE: Chronic Atypical Neutrophilic Dermatosis with Lipodystrophy and Elevated temperature; SAVI: STING-Associated Vasculopathy with onset during Infancy; NOMID: Neonatal-Onset Multisystem Inflammatory Disease; HC: healthy controls; MSA-neg: subgroup of JDM testing negative for myositis-specific autoantibodies.

**Figure S3**. NF-κB ratio in JDM with elevated IRG-S and by MSA groups versus other conditions

Median and interquartile ranges shown. Dotted horizontal lines represents range of healthy controls and NOMID, distinguishing different interferonopathy groups (26). *: p <0.0001.

CANDLE (n=11), SAVI (n=7), NOMID (n=18), HC (n=26) are included and compared with:

A. JDM with IRG-S above healthy control range or >49 (n= 26) (22)

and

B. MSA groups with IRG-S above healthy control range: Anti-TIF1 (n=9), anti-NXP2 (n=4), anti-MDA5 (n=7) autoantibodies, and MSA negative (n=5). All of the MSA groups are significantly (p<0.05) lower than NOMID and HC.

Abbreviations: NF-κB: nuclear factor kappa B; JDM: juvenile dermatomyositis; IRG: interferon-regulated gene; IRG-S: interferon-regulated gene score; MSA: myositis-specific autoantibody; CANDLE: Chronic Atypical Neutrophilic Dermatosis with Lipodystrophy and Elevated temperature; SAVI: STING-Associated Vasculopathy with onset during Infancy; NOMID: Neonatal-Onset Multisystem Inflammatory Disease; HC: healthy controls; TIF1: subgroup of JDM with anti-TIF1 myositis-specific autoantibodies; NXP2: subgroup of JDM with anti-NXP2 myositis-specific autoantibodies; MDA5: subgroup of JDM with anti-MDA5 myositis-specific autoantibodies; MSA-neg: subgroup of JDM that had negative testing for myositis-specific autoantibodies.

**Figure S4**. IFNγ ratio in JDM with elevated IRG-S and by MSA groups versus other conditions


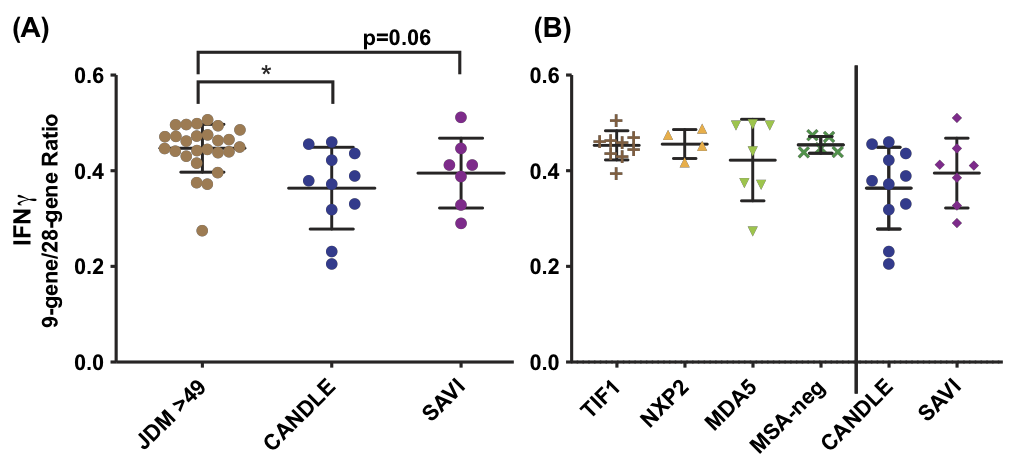


Median and interquartile ranges shown. *: p <0.005.

CANDLE (n=11), SAVI (n=7) are included and compared with:

A. JDM with IRG-S above healthy control range or >49 (n= 26) (22)

and

B. MSA groups with IRG-S above healthy control range: Anti-TIF1 (n=9), anti-NXP2 (n=4), anti-MDA5 (n=7) autoantibodies, and MSA negative (n=5).

Abbreviations: JDM: juvenile dermatomyositis; IRG: interferon-regulated gene; IRG-S: interferon-regulated gene score; MSA: myositis-specific autoantibody; CANDLE: Chronic Atypical Neutrophilic Dermatosis with Lipodystrophy and Elevated temperature; SAVI: STING-Associated Vasculopathy with onset during Infancy; TIF1: subgroup of JDM with anti-TIF1 myositis- specific autoantibodies; NXP2: subgroup of JDM with anti-NXP2 myositis-specific autoantibodies; MDA5: subgroup of JDM with anti-MDA5 myositis-specific autoantibodies; MSA-neg: subgroup of JDM that had negative testing for myositis-specific autoantibodies.
